# Post-Mortem Treatment of the Dental Pulp

**Published:** 1893-02

**Authors:** Otto Arnold

**Affiliations:** Columbus, O.


### Post-mortem Treatment of the Dental Pulp.

BY OTTO ARNOLD, D.D S., COLUMBUS, O.

Read before the Ohio State Dental Society, December, 1892.

For many years it has been the custom in conservative surgery to remove dead from living tissue, that the latter may not be encumbered by disease at the triumph of the former. This has been for a long time the accepted practice, and I know of no evidence at present, indicating a revolution in custom.

In conservative dentistry the same law prevails; not because of a desire to imitate—not at all—, but for the reason that it is known to be philosophical and in accord with natural principles and laws, therefore it is scientific. Science implies comprehensive information, complete knowledge, ascertained facts about anything, hence—explainable. Speculative phenomena are not truly scientific, unless their cause and effect may be accounted for.

In the generally accepted treatment for the devitalization of a dental pulp, and the subsequent management and preparation of the pulp canal for its permanent filling, it seems to me we have well-nigh reached perfection, theoretically at least, and that the principles involved convey comprehensive and philosophical conviction.

To more clearly state my proposition I will illustrate by a brief analysis. A case presents for your consideration, in which is involved an exposed tooth pulp. For reasons satisfactory to yourself, devitalization of the pulp is agreed upon. Believing in arseneous acid as a devitalizing agent, you proceed to apply it, observing the usual precautions throughout your manipulations, dismissing your patient for two, six or twenty-four hours, as in your judgment seems best. At the appointed time the cavity is unsealed and results are investigated. You discover no sensation in the pulp, and thus far are satisfied; accordingly, as anatomical relations are favorable or not, you conclude to extirpate the pulp at once or defer the operation for a more convenient season. After the pulp has been extirpated, you fill the canal with a suitable substance, either at once or later, using your judgment to the best interests of the case in hand.

There is nothing chimerical or speculative about the process, from beginning to end. It is the outcome of years of progressive experiment and clinical experience, and is accepted as a rational and conservative method wherever modern dentistry is known. It seems nothing short of assumption for any one to defend a system that has so thoroughly stood the test of time and given such beneficent results.

But we are now confronted with a proposition in practice, or a method, that is directly opposed to the principles of surgical science, which omits what we believe to be the most essential feature, viz: the removal of the dead pulp. I therefore choose to appear as an adversary of the latter, rather, than as a defender of a rational and accepted theory.

Briefly described, the details of the method are as follows: Cobalt, to which has been added eight per cent. of hydro-chlorate of cocaine, is applied to the exposed pulp and allowed to remain two or three days, then removed, the cavity cleaned from decay and disinfected. Then the coronal portion of the pulp is amputated by a large, sharp bur rapidly revolved in the engine hand-piece. The pulp chamber is then washed out with a one-tenth of one per cent. bichloride solution, and dried. Now a loosely-rolled cylinder or ball of tin or gold foil is placed into the pulp chamber, directly upon the pulp stump, and with a smooth burnisher revolving in the engine hand-piece, burnished firmly to place.

This in substance is the method that Dr. Herbst, of Germany, submits to the dental profession for approval and endorsement. How far he may succeed, depends in a great measure upon the susceptibility of individual members of the profession, to adopt, without investigation, doctrines that present irrational features wholly inconsistent with scientific facts. I venture, however, the assertion that conservative dentists of this country will be slow to retrace their steps and abandon methods which are definite and positive in results, for this German method, which promises nothing good definitely, but much evil indefinitely.

Chemically, arsenic and cobalt are practically alike, with different degrees of potency only; the terms may therefore be used synonymously. The addition of cocaine, to either, may modify pain, but adds no prophylactic properties whatever. Now, if there were any doubts concerning the destructive action of arsenic upon organized tissue, if it acted merely as an anæsthetic, and only temporarily suspended sensibility in the pulp, we might have reason to expect a return of function in the future, in which case there would be only a change in environment, provided, that during the period of vital suspension the cavity had been filled.

However uncertain we may be as to the manner in which disorganization takes place, we most certainly do know that, unlike Friar Laurence's potion given to Juliet, arsenic kills; and if there is any reorganization, it is accomplished only on the other side of Jordan, from whence we have no definite returns.

We also know, from abundant evidence, clinically—most of us, at least,—that a dental pulp subjected to arsenic is not rendered immune against septic and bacterial influences. If arsenic established immunity, there would have been no occasion to change the method practiced, more than fifty years ago, by Dr. Spooner and his confreres, who first employed arsenic for destroying tooth pulps, immediately following the process by filling, without removing the pulp, and using, indiscriminately, all substances by any of the then known methods, *and this was before the advent of the dental mallet.* But, as pericemental disturbances and alveolar abscesses subsequently appeared in most cases so treated, and failure upon failure followed sooner or later, it finally dawned upon these gentlemen that the post-mortem disposition of the pulp was an important factor, and proved beyond all doubt that while in its normal state it existed in perfect contentment—after death it made things very uncomfortable, unless appropriate obsequies and a foreign burial place were provided.

I doubt not that in Dr. Spooner's practice many cases thus treated remained comfortable for a long time, and perhaps others continued so throughout the life of the individual. But extraordinary conditions do not establish rules, but exceptions. We can not lose sight of the natural agencies inherent in living tissues, and their power of combating and arresting destructive tissue metamorphosis. The processes of encystment and absorption are well-known types, often providing the only safeguards against rapid and extensive tissue degeneration. Most of us, perhaps, are familiar with unexplainable conditions at times, but these must all be classed as exceptions, and they furnish no reliable basis for rule in practice.

A few years ago, when pulp capping was more extensively practiced than now, the most unpromising cases sometimes survived the treatment and seemed to prosper indefinitely; when by accident perhaps the pulps were later discovered dead. And yet, the usual evidences of the true condition were never apparent, because nature kindly *tolerated* the abnormal state. But this is not the rule, and we should be grateful for the rescue, rather than to accept it as an inevitable sequence, and refrain from imposing upon nature too frequently.

Has there been nothing gained from these clinical facts? If

I know nothing more, I surely have learned to select my cases more carefully when I practice pulp capping.

Pulps often die from accidental causes in perfectly sound teeth, remain for a long time inert as it were, but ultimately assert their power, and must be removed for the benefit of health and comfort. We surely believe that a pulp within a sound tooth is more thoroughly protected from septic infection than one once exposed, and covered by a filling, no matter by what method it had been inserted. Air-tight fillings, of whatever material, do not render a dead pulp immune from bacterial infection. It must be removed; and just in proportion as this is thoroughly or partially done you induce the establishment of a condition that will generate trouble sooner or later.

There seems to be no definite rule governing the interregnum between the death of the pulp and the period when this fact is made manifest by subjective or objective symptoms, or both. Indeed, the prolonged immunity from discomfort that is sometimes experienced after the immediate or local disturbance following violence has subsided, likewise the immunity sometimes following pulp capping, furnish the only basis of hope for advocates of the Herbst or similar methods. This variation in the course of time is due to systemic conditions, depending upon the inherent power of resistance or the *vis vitæ.* But more often symptoms of pulp dissolution rapidly appear, and prompt surgical interference is demanded to arrest the evil.

Decomposition of unsterilized dead tissue is an inevitable consequence, whether it be of a part or the whole of the body. The same may be modified in character, however, by the influence of specific bacteria.

Pathologists have, for a long time, recognized the presence, in blood-vessels of cadavers, of a gas which did not seem attributable to the ordinary post-mortem decomposition. This has given rise to much speculative theorizing in the past, and has recently led to the discovery of a micro-organism that seems destined to clear the mystery.

This micro-organism—"Bacillus aerogenes capsulatus," so-called,—was discovered and cultivated in the pathological laboratory of Johns-Hopkins University and Hospital, and is reported fully in their Bulletin for July and August. Experiments with cultures upon live rabbits give interesting and valuable results that may be significant to the student of dental pathology; viz: in none of the animals inoculated was there any evidence manifested of the gaseous condition (or presence of bacteria) during life, but shortly after they were killed, by violence of course, gas and bacilli were rapidly developed. In one case only—that of a pregnant rabbit—the injection proved fatal, which at the autopsy was found attributable to the remarkable fact that "two of the embryos in the uterus were already dead when the injection was made.

Upon the strength of such evidence it does not seem altogether unreasonable to recognize a pathological analogy between dead tooth pulps and dead embryos, and we might safely attribute the presence of gas in the dental canal at times to the same agencies. It may, at least, help to solve the problem in those cases that develop most rapidly, and to my mind the difference is only in extent.

At any rate a dead tooth-pulp is a disturbing element to the adjacent tissues, inasmuch as it becomes a focus for the colonization of bacilli that multiply rapidly and carry destruction in their wake. When this occurs, nature's sanitary forces, the leucocytes, muster in large numbers and hasten to the spot in battle array, and a combat begins. Leucocytes attack bacilli; in turn becoming phagocytes they devour bacilli and keep up the good work. But, alas! bacterial fecundity is too prolific, and the ranks are recruited more rapidly than they are devoured, and it is only a question of time when our friends are overpowered and the enemy is victorious.

My friends, it is against nature to act as a burying place for dead tissue. At best she may, by her power of resistance, defer for a time the climax, and tolerate the imposition. If you would seek to lay a foundation for success in tooth conservation, let your motto be: "Dead pulps must be removed! Take no chances by imposing too much upon nature."

I am fully aware of the difficulties we often encounter, and of the obstacles that are sometimes insurmountable, and render it physically impossible to remove the pulps completely in all cases presented. The writer has toiled often and long in search of an opening into buccal roots of superior molars, and had only his labor for the pains. Again, at the last moment, when about to give up in despair, I have found a long-searched-for canal. I need not describe, here, the obstacles that often prevent a thorough removal of the pulp from anterior root canals in inferior molars. But there must be a standard of perfection, and I insist upon honest endeavors to reach it. We can not at this time go backward a half century or welcome obsolete methods. Science is progressive, and in dentistry we propose to keep apace.

It seems hardly necessary to rehearse before this audience methods and processes in detail for removing the dental pulp. If there are any seeking light in this direction, I can do them no better service than to commend, for their reading, an article in the October *Cosmos*, one of a series on filling teeth, by Dr. Ottolengui, wherein the subject is treated most thoroughly and rationally.

However, as it would be inconsistent to ignore, altogether, the subject as implied in the title of this paper, I will, at least, note some of the principles that I consider important in the treatment —or the removal if you please—of devitalized pulps, and some of the aids that have benefited me in this kind of work.

The first condition I desire is cleanliness within the cavity made by decay, and about the instruments used. The next step is to secure access to the canals, even at the expense of good tooth structure. Manipulative ability is always impaired by obstruction of light and approach. Access obtained, proceed to introduce into the canal a barbed broach which has been sterilized by dipping it in creosote, passing it along between the wall of the canal and the pulp tissue. When the apex has been reached, gently rotate the broach until the sense of touch indicates to your mind that the tissue has become entangled upon the instrument, then gently withdraw it with the pulp adherent.

In all reasonably straight roots with symmetrical canals, wherein the pulp tissue has not reached an advanced state of disintegration or putrefaction, this will usually accomplish a complete removal of the pulp.

In posterior teeth, and particularly in the anterior canals of inferior molars, I have found Donaldson's adjustable broach-holder a valuable aid. In all difficult cases I know of no better aid to your manipulations than an inexhaustible supply of patience, accompanied by a determination to succeed in the undertaking.

When, however, all has been done, and your manipulative resources have become exhausted, with failure to entirely extricate the pulp, you may resort to some of the pepsin preparations, packing the canal full, frequently changing the same, which in time may digest the dead tissue left in the canal.

Or, as a last resort, leave the canal alone, filling the crown cavity with an easily removable temporary stopping and await developments. When pericemental inflammation or symptoms of the same appear, the canal may be reopened, treated antiseptically, and subseqnently closed permanently. Taking it for granted, however, that in the multiple-rooted teeth all canals had been previously filled from which the pulps were removed.

The consideration of treatment for putrescent canals, and the more extensive lesions possible, primarily, from pulp decomposition, have purposely been avoided. The writer aiming chiefly to point out, with the strongest emphasis at his command, the importance of removing dead tooth pulps.

